# The Bergen 4-day treatment for social anxiety disorder: a pilot study

**DOI:** 10.1186/s12888-024-05607-4

**Published:** 2024-02-21

**Authors:** Bjarne Hansen, Thorstein Olsen Eide, Marie Aaslie Reiråskag, Kristian August Tjelle, Stian Solem, Kristen Hagen

**Affiliations:** 1 Bergen Center for Brain Plasticity, Haukeland University Hospital, Bergen, Norway; 2 Center for Crisis Psychology, Faculty of Psychology, University of Bergen, Bergen, Norway; 3 Department of Psychiatry, Molde Hospital, Møre Og Romsdal Hospital Trust, Molde, Norway; 4 Department of Health and Nursing Sciences, University of Agder, Kristiansand, Norway; 5 Department of Psychology, Norwegian University of Science and Technology, Trondheim, Norway; 6 Department of Mental Health, Norwegian University of Science and Technology, Trondheim, Norway

**Keywords:** Social anxiety disorder, Intensive treatment, Exposure, B4DT, CBT

## Abstract

**Background:**

Few studies have examined the use of concentrated and intensified cognitive behaviour therapy for treating social anxiety disorder (SAD). The aim of this study was to examine the feasibility of the Bergen 4-Day Treatment (B4DT) for treating SAD.

**Methods:**

This study adopted an open trial design without a control group. Thirty consecutively referred patients who were diagnosed with SAD were treated and assessed at pre-treatment, at post-treatment, and at the 3-month follow-up. The Liebowitz Social Anxiety Scale was used to assess symptoms of SAD; the Generalized Anxiety Disorder-7 scale was used to assess anxiety symptoms; and the Patient Health Questionnaire-9 was used to assess symptoms of anxiety and depression. The Client Satisfaction Questionnaire-8 was administered posttreatment.

**Results:**

Overall, patients reported a high level of satisfaction with the B4DT. Large effect sizes were observed for symptoms of SAD (*d* = 1.94–2.66) and for the secondary outcomes, i.e., generalized anxiety (*d* = 0.86–0.99) and depression (*d* = 0.62–0.83). The remission rate was 55.2% at follow-up, while the treatment response rate was 89.7%.

**Conclusions:**

The B4DT is a promising treatment approach for patients with SAD. In the future, controlled trials should be performed to compare the efficacy of this treatment approach with standard outpatient treatment. Practical consequences, policy implications, and suggestions for future research are discussed herein.

## Introduction

Social anxiety disorder (SAD) is characterized by intense fear and anxiety regarding social situations wherein one might be negatively evaluated by others; therefore, individual with SAD feel that social situations are likely to induce anxiety and hence is avoided or endured [1]. The prevalence of SAD is estimated to be 13%, making it the most prevalent anxiety disorder after specific phobias [2]. Patients with SAD report substantial functional impairment, specifically in terms of work, studies and social life [3]. Comorbidity is common in patients with SAD, with estimates in previous studies ranging from 55% [3] to 90% [4]. The most common comorbid disorders are depression and other common anxiety disorders [4]. SAD tends to be a chronic disorder when it is untreated, and the prevalence of spontaneous remission is low [5, 6].

Cognitive behavioural therapy (CBT) is considered the “gold standard” treatment for patients with SAD [7]. Several meta-analyses have demonstrated that CBT is an effective treatment option for SAD, as indicated by large effect sizes (*g* = 0.80) on disorder-specific outcomes compared with what is observed in control groups (mostly waitlists) [8, 9]. The within-group effect sizes of CBT for SAD in routine clinical care were g = 1.26 at posttreatment and 1.41 at follow-up. For symptoms of depression, the effect sizes were 0.65 at posttreatment and 0.60 at follow-up [10]. Moderate-to-large effect sizes have also been found in the reduction of trait anxiety (*g* = 0.65) [8]. A previous study showed that the mean remission rates for patients with SAD who completed CBT were 40.4% at posttreatment and 45.4% at follow-up [11]. Pharmacological treatment with selective serotonin reuptake inhibitors (SSRIs) has also been shown to be an effective treatment option compared with waiting list controls (SMD = 0.91) and can be recommended for patients who decline CBT [9].

The development of intensified and concentrated CBT formats has been achieved by reducing either the number of sessions or the time intervals between sessions [12]. Existing studies on concentrated or intensified CBT for anxiety disorders have revealed treatment outcomes comparable to those of standard CBT [13, 14]. Intensifying CBT seems to be associated with lower attrition rates [12] and quicker responses [14]. Furthermore, intensifying CBT might also contribute to a greater reduction in comorbid symptoms of depression symptoms among patients with SAD [13]. However, this type of treatment might be slightly more demanding for patients [15].

Research on intensified group CBT (*n* = 26) for SAD has demonstrated promising results compared to waitlist groups [16]; the within-group effect sizes of intensified group CBT ranged from 0.56 to 0.81 for symptoms of SADs and from 0.14 to 0.39 for depression symptoms. In another study that compared standard individual CBT to intensified group CBT for patients with SAD, standard CBT was shown to be associated with a slightly greater treatment response than what was observed in the intensified group CBT [17]; however, at the 5-year follow-up, there was no significant difference between the treatment conditions [18]. Notably, few studies have examined the use of concentrated and intensified CBT for SAD; therefore, further research is necessary [13].

The Bergen 4-day treatment (B4DT) is a form of concentrated CBT, and it has been shown to be a promising treatment option for obsessive‒compulsive disorder (OCD) [19–25] and panic disorder [26–29]. To date, no studies have examined the effectiveness of the B4DT for treating SAD. The B4DT is delivered to groups of 3–6 patients with a 1:1 ratio between patients and therapists. The treatment is delivered across four consecutive days in a public outpatient clinic.

The standard outpatient model with weekly sessions is based more on traditional methods than research. A longer time between sessions could stall progress and increase attrition. Guidelines also often recommend a stepped care approach for treating anxiety disorders, but it is unclear whether this approach actually improves recovery rates [19]. Patients may prefer a quicker reduction in symptoms; furthermore, intensive treatment is an acceptable strategy for achieving faster symptom reduction and should therefore be considered an alternative to standard weekly treatment [14, 15].

The aim of this study was to examine the feasibility of the B4DT for SAD patients. We hypothesized that the B4DT would be associated with a significant reduction in symptoms of SAD.

## Method

### Participants and procedure

**Fig. 1:**
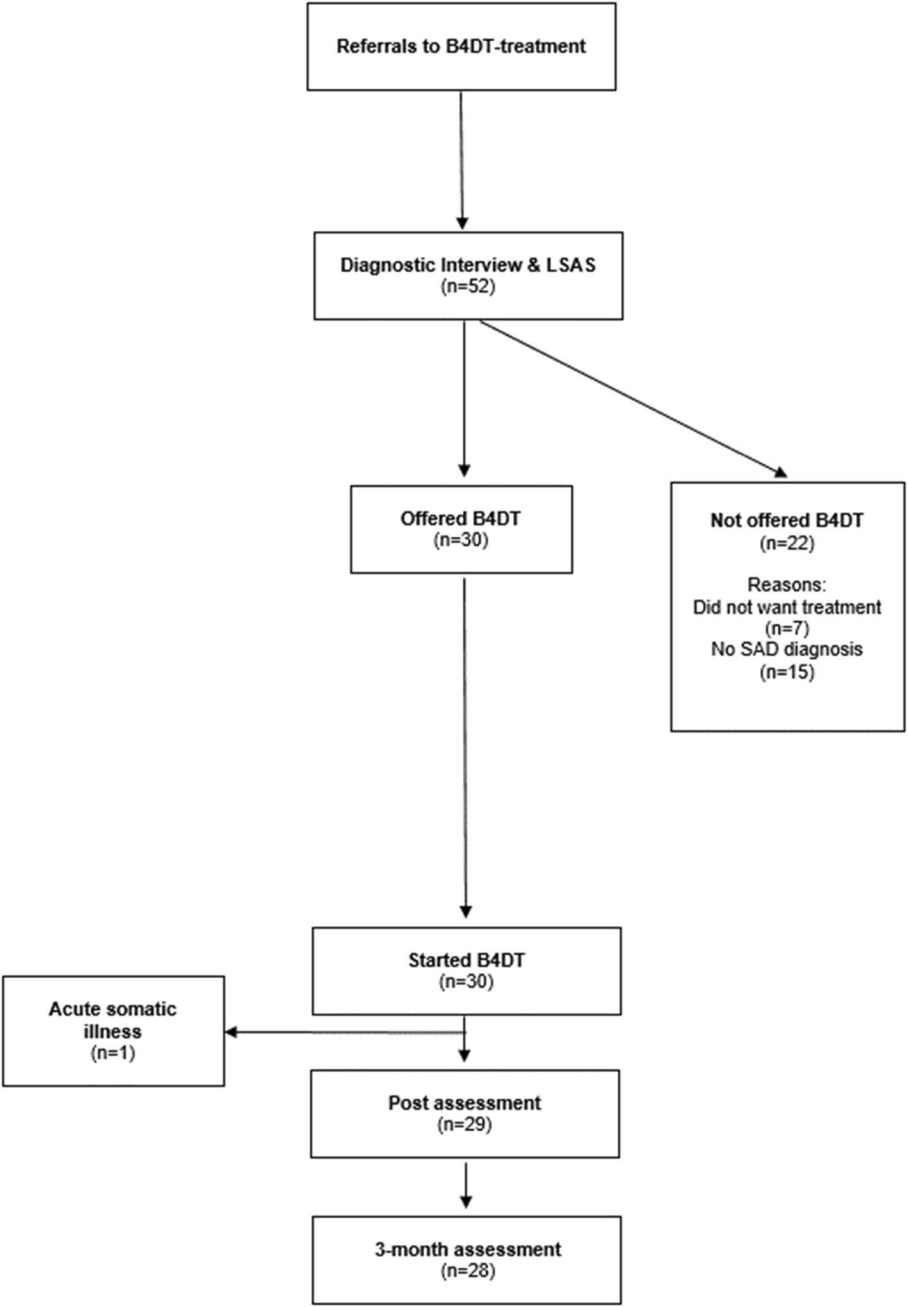
Participant flow chart

The study used an open trial design without a control group. The study was conducted at an outpatient clinic in Molde. This clinic is part of the specialist healthcare in Helse Møre & Romsdal Hospital Trust in Norway. This study entailed a naturalistic quality assessment of the treatment outcome of patients treated with the B4DT for SAD. The patients were recommended for the B4DT treatment either by their general practitioner or by other clinicians in the public health care system. Seven patients declined treatment, and 15 patients did not meet the criteria for SAD. Therefore, the intention-to-treat sample consisted of thirty consecutively recruited patients. Figure 1 shows the flow chart of patient inclusion. The inclusion criteria were as follows: a diagnosis of generalized social anxiety, and providing written informed consent. The exclusion criteria were: active substance abuse, were actively suicidal, psychotic, bipolar in an unstable phase, or did not speak Norwegian.

The data collection started in 2019 and finished in 2022. All patients were diagnosed before treatment via a structured clinical interview that was administered by a trained and experienced clinician. All patients who met the diagnostic criteria for SAD were offered treatment.

A summary of the sample’s demographic information can be found in Table 1. The majority of the patients were female (73.3%, *n* = 22), and the mean age was 26.5 years (*SD* = 7.26, range = 18–45). The patients had suffered from SAD for a mean of 8.10 years (*SD* = 5.40), with a range of 1 to 24 years. Of the patients treated, 73.1% (*n* = 22) had previously received psychiatric treatment for SAD. A total of 63.3% (*n* = 19) of the participants were employed or enrolled in educational programs, while the remainder were receiving social welfare benefits.

**Table 1:** Sample characteristics at pre-treatment (*N* = 30)

Variable	*M* (*SD*)	N (%)
Female sex		22 (73.1)
Age	26.50 (7.26)	
Duration of the disorder (years)	8.10 (5.30)	
Previous psychiatric treatment		22 (73.1)
In a relationship		13 (43.3)
University/college education		11 (36.7)
Working/Studying		19 (63.3)
Comorbidity		20 (66.7)
Depression		14 (46.7)
Panic disorder		7 (23.3)
GAD		6 (20%)
Psychotropic medication		9 (30.0)
SSRI		7(23.3)

*GAD* generalized anxiety disorder, *SSRI* selective serotonin reuptake inhibitor

In total, 66.7% (*n* = 20) of the patients had at least one comorbid psychiatric disorder. The most common comorbid disorders were depression (46.7%, *n* = 14) and panic disorder (23.3%, *n* = 7). The patients who were using psychotropic medication were instructed to maintain a stable dose for four weeks before starting the B4DT. The most commonly used psychotropic medications were antidepressants (23.3%, *n* = 7).

### Treatment

The B4DT for SAD was developed based on the B4DT model for OCD [19, 20] and the B4DT model for panic disorder [26]. A key element of this treatment approach involves exposing patients to specific situations – not only to induce distress but also to facilitate the acquisition of new strategies for effectively managing emotional discomfort. Another central element of the B4DT is the use of the LEaning in Technique (LET). During exposure, patients frequently employ self-restraint through safety behaviours or mental regulation. The B4DT helps patients identify their safety behaviours and encourages them to relinquish these behaviours and “lean into the anxiety." This marks a pivotal decision point that is characterized by continuous demonstration, practice, and monitoring of the LET. Patients are guided by questions such as "To what extent are you holding back?" and "Are you willing to let go?". The emphasis lies in how the exposure is conducted. The treatment emphasizes the application of the LET during exposure exercises. Patients are taught that it is more efficacious to execute a simple exposure task accurately, which entails full engagement without employing safety behaviours, rather than approaching a more complex task with safety-seeking strategies. The progression to more challenging and intricate tasks should be contingent upon the patient's comprehensive grasp of the correct application of the LET during exposure. Following each session, evaluations are performed to assess the degree to which patients employ the LET and to assess their proficiency in altering established patterns (Table 2).

**Table 2:** Outline of the treatment

Time	Day 1	Day 2	Day 3	Day 4
0830–0930		Repetition Psychoeducation and Introduction to LET	Exposure treatment based on LET individually and/or in group	Psychoeducation: How to integrate the treatment principles in their life
0930–1030	Psychoeducation	Exposure treatment based on LET individually and/or in group		Summarize lessons learnt and planning for the time after the group
1030–1130				
1130–1230	Planning of individualized exposure tasks and setting goals for the treatment	Summarizing learning experiences and eating lunch with the group	Summarizing learning experiences and eating lunch with the group	
1230–1330		Exposure treatment based on LET individually and/or in group	Exposure treatment based on LET individually and/or in group	
1330–1430				
1430–1530				
1530–1630		Emphasis on independent integration of treatment principles into everyday life (not therapist assisted)	Psychoeducation for families/significant others	
1630-			Emphasis on independent integration of treatment principles into everyday life (not therapist assisted)	

On the first day, patients attend a thorough psychoeducational session (approximately 4 h) that provided in-depth information on the features of SAD, factors contributing to its persistence, and the guiding principles of treatment. Additionally, the patients compile a list of exposure tasks to be undertaken in the following days. The second and third days (lasting approximately 7 h) are specifically devoted to therapist-assisted exposure therapy, which is customized based on to the patient’s needs. Patients are exposed in vivo to social situations that typically elicit avoidance or intense anxiety.

Group sessions are conducted to practice activities such as giving presentations, being the focus of attention, and speaking assertively. Furthermore, individual exposure tasks, such as simulating a job interview, engaging in small talk, answering phone calls, and participating in shopping scenarios, are assigned to address individual fears. There are regular group meetings scheduled where the patients can report their progress and receive feedback from other members of the group. The primary emphasis during the exposure days is on practising the LET and ensuring that patients had many opportunities to practice it in various settings. Once they master the technique, patients are actively encouraged to confront challenging situations without delay and to prioritize practising in situations that would yield the most significant impact on their daily life.

The fourth day, spanning approximately 3 h, is focused on maintaining the progress achieved by the patients and introducing the principles of relapse prevention. Collaboratively, the patients and the therapist devise an individualized exposure plan for the next three weeks, thus ensuring continued growth and stability.

### Therapists

The treatment team consisted of eight therapists, all of whom were either clinical psychologists or psychiatrists. The two group leaders had 10 and 7 years of experience and were certified as group leaders for the B4DT program. The remaining therapists were certified as B4DT therapists, indicating that they had participated as therapists in at least three treatment groups. Throughout the treatment process, regular meetings were held for therapists to discuss progress and challenges. The group leaders provided supervision and assistance whenever necessary. At the end of every treatment, all the therapists completed a form addressing whether the treatment had been conducted according to the treatment manual. There were no indications of deviation from the treatment manual in this study.

### Assessment

Evaluations were conducted before treatment, 10 days after the conclusion of treatment, and at the 3-month follow-up. The patients completed self-report measures of depression, generalized anxiety, and client satisfaction using a secure online platform. The patients were reminded to complete the questionnaires at predetermined intervals via text messages. An independent assessor conducted the posttreatment and follow-up interviews.

### Measures

The Liebowitz Social Anxiety Scale (LSAS) [30] is a 24-item instrument developed for assessing the severity of SAD. The interview assesses 24 different social situations where patients are asked to rate both their fear and avoidance on a 0–3 Likert-type scale. Example items include “Going to a party”, “Meeting strangers”, “Speaking up at a meeting”, and “Giving a prepared oral talk to a group”. The total score ranges from 0–144, with a higher score indicating greater severity of SAD [30]. A total score of 60 has been identified as an optimal cut-off point for diagnosing SAD [31]. The LSAS has demonstrated reliability and validity, and it is sensitive to change [32].

The Generalized Anxiety Disorder-7 scale (GAD-7) [33] is a validated questionnaire designed to measure symptoms of generalized anxiety disorder. The GAD-7 comprises seven items that are answered on a four-point Likert scale (0 = not at all, 3 = almost every day). Example items include “Feeling nervous, anxious, or on edge” and “Worrying too much about different things”. Total scores on the GAD-7 range from 0 to 21, with corresponding interpretations: scores of 0–4 indicate "minimal anxiety," scores of 5–9 indicate "mild anxiety," scores of 9–14 indicate "moderate anxiety," and scores of 15–21 indicate "severe anxiety." The GAD-7 has been shown to exhibit acceptable reliability and validity [33].

The Patient Health Questionnaire-9 (PHQ-9; [34]) is a tool that assesses depression symptoms based on the DSM-IV criteria. The PHQ-9 includes nine items that are answered on a four-point Likert-type scale (0 = not at all, 3 = almost every day). Example items include “Feeling down, depressed, or hopeless” and “Feeling bad about yourself – or that you are a failure or have let yourself or your family down”. Total scores on the PHQ-9 range from 0 to 27, with corresponding interpretations: scores of 0–4 indicate "none," scores of 5–9 indicate "mild," scores of 10–14 indicate "moderate," scores of 15–19 indicate "moderate to severe," and scores of ≥ 20 are considered "severe." The PHQ-9 has been shown to exhibit acceptable reliability and validity [35].

The Client Satisfaction Questionnaire-8 (CSQ-8) [36] is an 8-item questionnaire that employs a 4-point Likert scale to measure client satisfaction with health services. Scores on the CSQ-8 range from 8 to 32, with higher scores reflecting a greater level of satisfaction. The questionnaire has been shown to exhibit favourable psychometric properties [36].

The Mini International Neuropsychiatric Interview (MINI) [37] was utilized for the diagnosis of SAD and comorbid disorders during the screening process. The MINI is a structured interview that is specifically designed to assess Axis-1 DSM-IV disorders. The Norwegian version of the MINI has been shown to exhibit favourable psychometric properties [38].

### Statistical analysis

This study included a relatively small number of missing data. For the LSAS assessment, only one patient had missing scores (at the 3-month follow-up). Regarding the self-reported data, 4.98% of the responses were missing across all assessment points. The expectation maximization (EM) method was performed in SPSS version 29 to impute missing data. This method was utilized because the dataset had less than 25% missing data, and the missing data were found to be missing at random based on Little's MCAR test (*x*^2^ [27] = 29.47, *p* = 0.34) and Kolmogorov–Smirnov test indicated that the data were normally distributed. When these conditions are met, EM is a reliable approach for imputing missing data [39].

Repeated-measures ANOVA was used to examine changes in symptoms from pretreatment to posttreatment and follow-up, with scores on the LSAS, GAD-7, and PHQ-9 as dependent variables. The Greenhouse–Geisser correction was used when Mauchly’s test of sphericity was significant. For post hoc analysis, Bonferroni correction was used.

Based on recommendations from previous studies, remission was defined as a score of 30 or lower on the LSAS [31, 40–42]. Treatment response was defined as a reduction of at least 31% in the total LSAS score [41, 43].

## Results

One patient dropped out of the treatment because of acute somatic illness, and all the statistical analyses were conducted without data from this patient. All the other patients completed the treatment, yielding a completion rate of 97%. All completers were assessed posttreatment, and all patients except one were assessed at the 3-month follow-up (93%).

There was a significant reduction in symptoms of SAD over time (Table 3), *F*(1.86, 52.18) = 137.44, *p* < 0.001, *µ*_p_^2^ = 0.83. More specifically, there were significant reductions in symptoms of SAD from pretreatment to posttreatment (*d* = 1.94, *p* < 0.001), from pretreatment to follow-up (*d* = 2.66*, p* < 0.001), and from posttreatment to follow-up (*d* = 0.58*, p* = 0.007). The mean LSAS score of at pretreatment was 90, which was well above the suggested clinical cut-off value of 60; however, the mean LSAS scores at posttreatment and follow-up were 47 and 33, which were below the cut-off value.

**Table 3:** Results (*M* and *SD*) for the primary and secondary outcome measures (*N* = 29)

				Cohen’s* d*	
Variable	Pretreatment	Posttreatment	Follow-up	Pre-post	Pre-follow-up
LSAS	90.24 (20.64)	46.62 (24.12)	33.08 (22.26)	1.94	2.66
GAD-7	10.07 (5.61)	5.34 (3.67)	5.92 (3.78)	0.99	0.86
PHQ-9	11.21 (5.94)	6.61 (5.03)	7.57 (5.79)	0.83	0.62
CSQ-8		29.22 (2.60)			

*LSAS* Liebowitz Social Anxiety Scale, *GAD-7* Generalized Anxiety Disorder-7, *PHQ-9* Patient Health Questionnaire-9, *CSQ-8* Client Satisfaction Questionnaire-8. Cohen’s *d* = *(M*_*pre*_* – M*_*post*_*)/SD*_*pooled*_

There was also a significant reduction in symptoms of generalized anxiety disorder over time (*F* (1.33, 37.10) = 20.72, *p* < 0.001, *µ*_p_^2^ = 0.43). More specifically, there were significant reductions in symptoms of generalized anxiety disorder from pretreatment to posttreatment (*d* = 0.99*, p* < 0.001) and from pretreatment to follow-up (*d* = 0.86*, p* = 0.001). However, there was no significant change from posttreatment to follow-up (*d* = 0.15*, p* = 0.910). At pretreatment, the mean level of anxiety symptoms was moderate, which was reduced to mild at posttreatment and follow-up.

The results also revealed a significant reduction in symptoms of depression over time (*F*(1.60, 44.83) = 28.88, *p* < 0.001, *µ*_p_^2^ = 0.51). More specifically, there were significant reductions in depression from pretreatment to posttreatment (*d* = 0.83*, p* < 0.001) and from pretreatment to follow-up (*d* = 0.62*, p* < 0.001). However, there was no significant change from posttreatment to follow-up (*d* = 0.17*, p* = 0.191). At pretreatment, the level of depression symptoms was moderate, which was reduced to mild at posttreatment and follow-up.

The patients reported a high level of satisfaction with the treatment (see Table 4), as indicated by a mean score of 29.22 (*SD* = 2.60) on the CSQ-8. The total score varied between 24 and 32, with the most frequent scores being 32 (24.1%), 30 (13.8%), and 31 (13.8%). A total of 74% of the patients described the quality of care as excellent, 100% of the patients reported that they would recommend the treatment to a friend, and 92% of the patients reported that the treatment had fulfilled “almost all” or “most of my needs”.

**Table 4:** Posttreatment scores on the Client Satisfaction Questionnaire-8

Item	Scale points			
	1	2	3	4
1. Quality of service	0	0	7	20
2. Kind of Service	0	0	11	16
3. Met needs	0	2	14	11
4. Recommend to a friend	0	0	1	26
5. Amount of help	2	1	6	18
6. Deal with problems	0	0	8	19
7. Overall satisfaction	0	0	8	19
8. Come back	0	0	7	20

The mean CSQ-8 score was 29.22 (2.60), the range was 24–32, and the median was 30 (possible range 8–32). *N* = 27

At posttreatment, 79.3% (*n* = 23) of the patients were classified as treatment responders, and 37.9% (*n* = 11) were in remission. At the 3-month follow-up, 89.7% (*n* = 26) of the patients were classified as responders, and 55.2% (*n* = 16) were in remission.

## Discussion

This study represents the first attempt to assess the efficacy of the B4DT in patients with SAD. The results demonstrated that the clinical outcomes were favourable, as there was a substantial reduction in symptoms of SAD. The treatment demonstrated large effect sizes when comparing symptoms between pretreatment and posttreatment individuals as well as when comparing symptoms between individuals at pretreatment and the 3-month follow-up. These findings suggest that the B4DT is a promising treatment option for individuals with SAD. Only one patient discontinued the treatment (due to acute somatic illness); therefore, there was a low dropout rate. The low dropout rate and high treatment satisfaction score indicate that the treatment was well received by patients. These results are consistent with previous research on the effectiveness of the B4DT in treating OCD [19–25] and PD [26–29]. The results of this study are also consistent with the findings of previous studies indicating that concentrated or intensified CBT is an effective treatment for anxiety disorders [13, 14]. Compared with previous studies on intensive group-based CBT for SAD [16, 17], which reported effect sizes ranging from 0.56 to 0.81, the current study reported larger effect sizes (*d* = 1.94–2.66). Previous studies investigating the effectiveness of standard CBT for the treatment of SAD have reported effect sizes ranging from *g* = 0.80 [8] to SMD = 1.19 [9] and SMD = 0.92 for each group [9]. While the comparisons suggest that the B4DT is a promising approach for treating SAD, it is essential to exercise caution when interpreting the effect sizes, as variations in study design and samples may account for disparities in effect sizes. Additionally, higher pretreatment scores can influence the effect size, potentially contributing to the larger effect size observed in this study.

According to previous meta-analyses, the average remission rates of SAD after CBT were 40% at posttreatment and 45% at follow-up [11]. In contrast, this study reported comparable remission rates posttreatment (38%) and higher remission rates at follow-up (55%). The disparity between posttreatment and follow-up could be attributed to the concentrated format of the treatment, suggesting that patients require time to apply and reinforce their skills and new behaviours. The treatment response rate at posttreatment was 79%, exceeding the response rate of 60% reported in previous studies for CBT [41]. These findings provide additional support for the B4DT as a promising treatment approach for SAD.

The current sample reported a significant decrease in secondary outcome measures, i.e., symptoms of depression and generalized anxiety. These large reductions are in line with previous findings on the B4DT for both PD [26–29] and OCD [19–25]. The effect sizes for depression were larger than those reported by Mörtberg et al. [16]. In comparison to a previous study on standard CBT that yielded an effect size of g = 0.65 for trait anxiety [8], the current study reported larger effect sizes for the reduction of generalized anxiety (*d* = 0.86–0.99). The larger effect size could be explained by the larger reduction in symptoms of SAD, which also affects secondary outcome measures.

The current study has limitations. It was an open trial conducted in a naturalistic setting and lacked a control group. Larger and controlled studies are needed. Additionally, this study did not assess long-term outcomes and self-reported symptoms of SAD.

Introducing intensive treatment formats in routine health care could lead to some challenges. Switching from a traditional model to sessions once a week is not necessarily supported by therapists’ beliefs and financial systems in health care. Therefore, financial systems may have to adapt to allow for intensive treatment. Regarding therapists’ beliefs, a possible advantage of intensive treatment is that it could be helpful for training therapists. Exposure treatment is underutilized and often conducted at too low an intensity, resulting in slower symptom reduction [15]. The therapist training, structure, and group format could increase adherence to the treatment protocol as new therapists work together with experienced therapists. The format also allows therapists to focus their attention on a small group of patients and allows for the possibility of conducting exposure exercises in multiple settings.

Future research should investigate the cost-effectiveness of standard vs. intensive treatment. The total number of sessions may be similar, and the recovery rates and long-term effects may also be similar [22, 23]. However, intensive treatment could be associated with quicker return to work and lower dropout rates, and it could help health services to reduce the size of waitlists [25]. The format should therefore be evaluated using randomized controlled trials. The intensive format also has advantages for research, especially for predictor studies, as the effect of confounding variables is reduced. CBT principles may also be used for the prevention of social anxiety disorder, but cost‒benefit evaluations are needed [44].

## Conclusion

This is the first study examining the feasibility of the B4DT for social anxiety disorder. The B4DT appears to be a promising approach for treating social anxiety disorder, as it yielded large effect sizes and high rates of treatment response and remission. The treatment was well accepted by patients, as they reported high treatment satisfaction. Larger and controlled studies further examining this approach for treating social anxiety disorder are warranted.

## Data Availability

The anonymized datasets used during the current study are available from the corresponding author upon reasonable request.
